# Generalized shrinkage *F*-like statistics for testing an interaction term in gene expression analysis in the presence of heteroscedasticity

**DOI:** 10.1186/1471-2105-12-427

**Published:** 2011-11-01

**Authors:** Jie Yang, George Casella, Lauren M McIntyre

**Affiliations:** 1 Department of Preventive Medicine, Stony Brook University, Stony Brook, NY 11794, USA; 2 Department of Statistics, University of Florida, Gainesville, FL 32611, USA; 3 Department of Molecular Genetics and Microbiology, University of Florida, Gainesville, FL 32611, USA; 4 The Genetics Institute, University of Florida, Gainesville, FL 32611, USA

## Abstract

**Background:**

Many analyses of gene expression data involve hypothesis tests of an interaction term between two fixed effects, typically tested using a residual variance. In expression studies, the issue of variance heteroscedasticity has received much attention, and previous work has focused on either between-gene or within-gene heteroscedasticity. However, in a single experiment, heteroscedasticity may exist both within and between genes. Here we develop flexible shrinkage error estimators considering both between-gene and within-gene heteroscedasticity and use them to construct *F*-like test statistics for testing interactions, with cutoff values obtained by permutation. These permutation tests are complicated, and several permutation tests are investigated here.

**Results:**

Our proposed test statistics are compared with other existing shrinkage-type test statistics through extensive simulation studies and a real data example. The results show that the choice of permutation procedures has dramatically more influence on detection power than the choice of *F *or *F*-like test statistics. When both types of gene heteroscedasticity exist, our proposed test statistics can control preselected type-I errors and are more powerful. Raw data permutation is not valid in this setting. Whether unrestricted or restricted residual permutation should be used depends on the specific type of test statistic.

**Conclusions:**

The *F*-like test statistic that uses the proposed flexible shrinkage error estimator considering both types of gene heteroscedasticity and unrestricted residual permutation can provide a statistically valid and powerful test. Therefore, we recommended that it should always applied in the analysis of real gene expression data analysis to test an interaction term.

## Background

The regulation of gene expression starts when a cell's DNA is transcribed into mRNA. The simultaneous expression profiles of many genes under different circumstances can provide insight into physiological processes. Using modern technologies in gene expression experiments such as oligonucleotide arrays [1], and cDNA spotted arrays [2], many scientists have made novel discoveries about complex biological processes of yeast [3,4], drosophila [5], mice [6], humans [7], and other species. Recently one such study also included RNA-seq [8]. Statistical methodologies and issues involved in microarray data analysis have been widely reviewed [9-12], and it is expected that many of the same issues will need to be addressed with RNA-seq.

The analysis of variance (ANOVA) model is a popular statistical modeling method for the analysis of microarrays. Since its introduction by Kerr *et al*. [13], it has been extensively examined for use in this setting [14-21]. Kerr *et al*. constructed an ANOVA model that included the gene effect as a fixed effect. This model assumes identically and independently distributed residual errors across genes. The advantage of this model is that the large number of genes involved in a microarray experiment results in huge degrees of freedom for the error estimate, which can lead to a very powerful test. However, the common assumption of homoscedasticity may not hold true in this setting [22]. One alternative is to use an ANOVA model for each gene, but the resulting test statistics from gene-specific models may have limited power because the biological sample size for each gene in a microarray experiment is usually small.

To address this problem of limited power, researchers have proposed other methods for obtaining more information across genes, ranging from a simple equal-weighted average of a gene-specific error estimate and the global average of all gene-specific error estimates (*F*_2 _statistic proposed by Wu *et al*. [19] to empirical Bayesian modeling of all gene-specific errors [23-26]. Other variations [27-29] used different variance modeling strategies to address the heteroscedasticity problem, but no clear winner has emerged [30]. Huang and Liu [31] extended the test statistics proposed by Cui *et al*. [28] by assuming a normal distribution on the mean and then deriving an empirical Bayes likelihood ratio test. The resulting test statistic shrinks both the mean and variances.

In addition to the problem of *between*-gene heteroscedasticity, we must also be concerned with *within*-gene heteroscedasticity. For example, in the study of simple differential gene expression between a treatment group and a control group, the variance in the treatment arm may differ from that in the control arm. Some approaches to this problem include a general Bayesian framework to model heteroscedastic error in a single generalized linear mixed model setting [32] and a structural model placed on the error variances specific to each gene and treatment combination [33].

As gene expression studies become more popular, the complexity of the experiment increases. Instead of only simple treatment and control experiments, two or more factor experiments are being conducted. This increase in experiment complexity has led to many scientific questions involving the hypothesis testing of an interaction between two factors. For example, testing a probe by genotype interaction can result in inferences about polymorphism in the probe, such as single nucleotide polymorphism (SNP) and insertion-deletion (indel) [34-37]; testing a probe by sex can imply that alternative splicing occurs between male and female subjects [38]; and in pharmacogenomic studies, testing the genotype-drug/treatment or genotype-disease interaction may be of interest [39]. Thus far, all the development of ANOVA methods for microarray studies has focused on tests of main effects.

Here, a generalized shrinkage estimator incorporating both within- and between-gene heteroscedasticities is developed (see Lehmann and Cesella [40] for a review of shrinkage estimation). In any given experiment, both within-gene and between-gene heteroscedasticity may exist; thus, taking these possibilities into account should lead to an improved test statistic. Moreover, given the increasing complexity of recent studies and the burgeoning interest in hypotheses that involve interactions, we focus on an improved shrinkage-based *F*-test for interaction terms.

## Methods

Here we develop new shrinkage estimates for the error term and show how to use these estimates to construct *F*-like statistics. We then estimate the null distribution of these statistics by using permutation tests.

### Shrinkage error estimators

Shrinkage error estimators pull individual error estimates toward shrinkage targets, with the amount of shrinkage depending on the variability of individual error estimates [28,40]. Let the gene-specific error estimates for all genes *i *and subgroups *k *be

$$\left({\hat{\sigma }}_{1,1}^{2},...,{\hat{\sigma }}_{1,K}^{2},...,{\hat{\sigma }}_{I,K}^{2}\right),$$

*i *= 1,...,*I, k *= 1,..., *K*, and let $\sigma_{i,k}^{2}$ be the true variance of gene *i *in group *k*. When the experimental design is balanced, ${\hat{\sigma }}_{i,k}^{2}$ is the residual mean square for gene *i *in group *k *and $\nu {\hat{\sigma }}_{i,k}^{2}∕\sigma_{i,k}^{2}\sim \chi_{\nu }^{2}$, where *ν *represents the degrees of freedom for the error estimates.

The choices of shrinkage targets in microarray data include the following:

1. Specific values for each gene-group combination

2. Gene-specific values that are the same across all other groups

3. Group-specific values that are the same across genes but different across groups

4. A single point representing the underlying common error

Correspondingly, these targets are correct when (1) there are both within-gene and between-gene heteroscedasticity; (2) there is only between-gene heteroscedasticity; (3) there is only within-gene heteroscedasticity; and (4) all error variances are identical. We now develop a generalized shrinkage error estimator using these four shrinkage targets.

Let $X_{i,k}\equiv \log{\hat{\sigma }}_{i,k}^{2}-m\sim \log\sigma_{i,k}^{2}+\log\chi_{\nu }^{2}∕\nu -m$, where *m *is the mean of log $\log\chi_{\nu }^{2}∕\nu$. Then using asymptotic normal approximation of *X*_*i*,*k*_, the distribution of *X*_*i*,*k*_s with different shrinkage targets for different gene *i *and group *k *combinations is

(1) $$\begin{array}{c} X_{i,k}|\theta_{i,k}\sim N\left(\theta_{i,k},\sigma^{2}\right)\\ \;\;\theta_{i,k}\sim N\left(\mu +\alpha_{i}+\beta_{k},\tau^{2}\right), \end{array}$$

where $\tilde{\theta }=\left(\theta_{1,1},...,\theta_{1,K,}...,\theta_{I,1},...,\theta_{I,K}\right),\;\;\tilde{\alpha }=\left(\alpha_{1},...,\alpha_{I}\right)$ represents the gene-specific mean differences, and $\tilde{\beta }=\left(\beta_{1},...,\beta_{K}\right)$ models different means with respect to different classes of the subgroups.

If *σ*^*2 *^and *τ*^2 ^are known, then the Bayes estimator of *θ*_*i*,*k *_under the squared error loss is [39]:

$$\theta_{i,k}^{B}=\frac{\sigma^{2}}{\sigma^{2}+\tau^{2}}\left(\mu +\alpha_{i}+\beta_{k}\right)+\frac{\tau^{2}}{\sigma^{2}+\tau^{2}}X_{i,k}.$$

Here, *σ*^*2 *^is the variance of log $\chi_{\nu }^{2}∕\nu$ and is known [28,40], but *τ*^2 ^is not known. However, the marginal distribution of *X*_*i*,*k *_can be used to create an empirical Bayes estimator of *τ*^2 ^and hence of *θ*_*i*,*k*_. Marginally, *X*_*i*,*k *_~ *N*(*μ *+ *α*_*i *_+ *β*_*k*_, *σ*^2 ^+ *τ*^2^),*i *= 1,..., *I, k *= 1, ...*K*, and, from this model, the least square estimates of $\mu ,\tilde{\alpha },\tilde{\beta },\hat{\mu },\tilde{\alpha },\hat{\beta }$, are the uniformly minimum-variance and unbiased estimators. Using the fact that

$$E\left(\frac{\left[IK-\left(I+K-1\right)-2\right]}{\Sigma {\left(X_{i,k}-\hat{\mu }-{\hat{\alpha }}_{i}-{\hat{\beta }}_{k}\right)}^{2}}\right)=\frac{1}{\sigma^{2}+\tau^{2}},$$

the empirical Bayes estimator for *τ*^2 ^is $\Sigma {\left(X_{i,k}-\hat{\mu }-{\hat{\alpha }}_{i}-{\hat{\beta }}_{k}\right)}^{2}∕\left[IK-\left(I+K-1\right)-2\right]-\sigma^{2}$.

Then, we can construct the positive-part empirical Bayes estimator [40]:

$$\begin{array}{c} \theta_{i,k}^{EB+}={\hat{X}}_{i,k}+{\left(1-\frac{\left[IK-\left(I+K-1\right)-2\right]\sigma^{2}}{\Sigma {\left(X_{i,k}-\hat{\mu }-{\hat{\alpha }}_{i}-{\hat{\beta }}_{k}\right)}^{2}}\right)}_{+}{\hat{X}}_{i,k}\\ {\hat{X}}_{i,k}=\hat{\mu }+{\hat{\alpha }}_{i}+{\hat{\beta }}_{k}, \end{array}$$

where(*x*)_+ _= *max*(*x*, 0). The generalized shrinkage error estimate for *σ*_*i*,*k *_can be obtained through exponentiating $\theta_{i,k}^{EB+}$ as follows:

(2) $${\tilde{\sigma }}_{Gen,i,k}^{2}=\exp\left(\theta_{i,k}^{EB+}\right).$$

Using a similar argument, the generalized shrinkage error estimator with the shrinkage target at each gene is

(3) $$\begin{array}{c} {\tilde{\sigma }}_{Gen-gene,i,k}^{2}=\exp\left(m+\hat{\mu }+{\hat{\alpha }}_{i}\right)\\ {}*\exp\left[{\left(1-\frac{\left[IK-\left(I-1\right)-2\right]\sigma^{2}}{\Sigma {\left(X_{i,k}-\hat{\mu }-{\hat{\alpha }}_{i}\right)}^{2}}\right)}_{+}\left(X_{i,k}-\hat{\mu }-{\hat{\alpha }}_{i}\right)\right], \end{array}$$

with the shrinkage target at each group is

(4) $$\begin{array}{c} {\tilde{\sigma }}_{Gen-grp,i,k}^{2}=\exp\left(m+\hat{\mu }+{\hat{\beta }}_{k}\right)\\ {}*\exp\left[{\left(1-\frac{\left[IK-\left(K-1\right)-2\right]\sigma^{2}}{\Sigma {\left(X_{i,k}-\hat{\mu }-{\hat{\beta }}_{k}\right)}^{2}}\right)}_{+}\left(X_{i,k}-\hat{\mu }-{\hat{\beta }}_{k}\right)\right], \end{array}$$

and with the shrinkage target at the common error, we have

(5) $$\begin{array}{c} {\tilde{\sigma }}_{Gen-ce,i,k}^{2}=\exp\left(m+\hat{\mu }\right)\\ {}*\exp\left[{\left(1-\frac{\left[IK-3\right]\sigma^{2}}{\Sigma {\left(X_{i,k}-\hat{\mu }\right)}^{2}}\right)}_{+}\left(X_{i,k}-\hat{\mu }\right)\right]. \end{array}$$

The shrinkage error estimator proposed by Cui *et al*. [28] shrinks the gene-specific error estimators toward their common corrected geometric mean. Specifically, the estimator for $\sigma_{i}^{2}$ is calculated as

(6) $$\begin{array}{c} {\tilde{\sigma }}_{Cui,i}^{2}=\exp\left(m+\frac{\Sigma X_{i}}{I}\right)\\ {}*\exp\left[{\left(1-\frac{\left[I-3\right]\sigma^{2}}{\Sigma {\left(X_{i}-\frac{\Sigma X_{i}}{I}\right)}^{2}}\right)}_{+}\left(X_{i}-\frac{\Sigma X_{i}}{I}\right)\right], \end{array}$$

where *X*_*i *_is the residual variance estimate from a gene-specific model, and *m *and *σ*^2 ^are the mean and variance of log $\frac{\chi^{2}K\nu }{K\nu }$. The underlying assumption for this estimator is that there is no between-gene heteroscedasticity, as this estimator shrinks every gene-specific error estimator toward one target. Therefore, it will overshrink the gene-specific error estimates when gene heteroscedasticity exists. In comparison, generalized shrinkage error estimators are flexible in terms of incorporating a different type of heteroscedasticity. Some degrees of freedom are used for incorporating the heteroscedasticity. However, the gain is that the error estimator is then closer to the underlying distribution and should lead to better performance of the resultant F-like test statistics as shown in the results section.

In formulas (2), (3), (5), and (6), *m *is the mean and *σ*^2 ^is the variance of a log-transformed chi-square random variable. The simulation-based approximate values of *m *and *σ*^2 ^can be found from Table 1 in work of Cui *et al*. [28]. Pounds [41] gave analytical expressions for these parameters and developed R code for the exact calculation. Here, the simulation-based approximate values were used.

**Table 1:** Results from raw data permutation

Restricted?	Data set	*F*_1_	*F*_2_	*F*_3_	*F*_*Cui*_	*F*_*Gen*_	*F*_*Gen-gene*_	*F*_*Gen-grp*_
YES	null-ce	5.05(0.07)	5.06(0.08)	5.12(0.17)	5.09(0.10)	5.09(0.10)	5.05(0.08)	5.11(0.10)
	null-gh	5.02(0.07)	5.13(0.16)	5.26(0.20)	5.03(0.07)	5.07(0.12)	5.03(0.07)	5.11(0.16)
	null-wgh	4.97(0.07)	4.96(0.09)	4.93(0.18)	4.99(0.08)	4.99(0.12)	4.96(0.09)	5.01(0.16)
	null-bgh	5.02(0.07)	4.99(0.17)	5.03(0.21)	5.02(0.07)	5.02(0.15)	5.01(0.09)	5.03(0.18)

NO	null-ce	5.10(0.07)	5.06(0.08)	5.06(0.08)	7.4(0.12)	5.15(0.09)	5.12(0.08)	5.08(0.08)
	null-gh	5.08(0.07)	5.12(0.16)	5.12(0.12)	7.4(0.09)	5.10(0.11)	5.07(0.10)	5.12(0.09)
	null-wgh	12.31(0.10)	7.56(0.10)	4.61(0.10)	17.37(0.14)	5.32(0.11)	5.07(0.09)	5.87(0.11)
	null-bgh	12.31(0.11)	6.63(0.17)	5.55(0.19)	15.68(0.12)	6.30(0.12)	6.10(0.11)	6.30(0.11)

CWER obtained from 1,000 permutations with the nominal significance level setting at 0.05, with standard errors in parentheses. Nine hundred simulation runs were performed to get empirical average CWER of all types of *F*-like test statistics.

### Shrinkage *F*-like statistics

To construct a statistic for the hypothesis test of no interaction between two fixed effects, the traditional *F*-test is simply the ratio of the mean square of the interaction term (MSI) and the mean square of residuals (MSE). This *F*-test, referred to as *F*_1 _[42], is $F_{1}=\frac{MSI}{MSE}=\frac{MSI}{{\hat{\sigma }}^{2}}$. The *F*_1 _test corresponding to a specific gene *i *is denoted by

(7) $$F_{1,i}=\frac{MSI_{i}}{{\hat{\sigma }}_{i}^{2}}.$$

The error variance estimator in this test uses data from only gene *i*. In oligonucleotide mi-croarray models, the degrees of freedom for the error estimate can be small because the sample size of RNA is usually small, and hence the power of *F*_1 _can be limited.

Following the method of constructing an *F*-test statistic given by Neter *et al*. [42], the gene-specific shrinkage *F*-like statistics for testing an interaction between two fixed effects can be obtained as

$$\begin{array}{ll} F_{Gen,i} & =\frac{MSI_{i}}{\Sigma_{k}{\tilde{\sigma }}_{Gen,i,k}^{2}∕K},\;\\ F_{Gen-gene,i} & =\frac{MSI_{i}}{\Sigma_{k}{\tilde{\sigma }}_{Gen-gene,i,k}^{2}∕K},\;\\ F_{Gen-grp,i} & =\frac{MSI_{i}}{\Sigma_{k}{\tilde{\sigma }}_{Gen-grp,i,k}^{2}∕K},\;\\ F_{Gen-ce,i} & =\frac{MSI_{i}}{\Sigma_{k}{\tilde{\sigma }}_{Gen-ce,i,k}^{2}∕K},\;\\ F_{Cui,i} & =\frac{MSI_{i}}{{\tilde{\sigma }}_{Cui,i}^{2}}.\;\\ & \; \end{array}$$

When the homoscedastic error assumption is true, the pooled variance estimator, ${\hat{\sigma }}_{pool}^{2}$, can be used to construct an *F*-like statistic. For a balanced design, the pooled variance estimate is the average of all gene-specific error estimates. This statistic is denoted by *F*_3 _using the same notation used by Cui and Churchill [22], who also introduced another shrinkage-type *F *statistic, *F*_2_, which can also borrow information across genes when estimating the residual variances. The statistic *F*_2 _uses an equal-weighted average of a gene-specific error estimator ${\hat{\sigma }}^{2}$ and ${\hat{\sigma }}_{pool}^{2}$. The definitions of *F*_2,*i *_and *F*_3,*i *_are

$$\begin{array}{c} F_{2,i}=\frac{MSI_{i}}{0.5{\hat{\sigma }}_{i}^{2}+0.5{\hat{\sigma }}_{pool}^{2}},\\ F_{3,i}=\frac{MSI_{i}}{{\hat{\sigma }}_{pool}^{2}}. \end{array}$$

### Permutation tests

For the proposed generalized shrinkage *F*-like test statistics, the null distributions are not known named distributions. Therefore, an empirical approach such as a permutation test can be used to estimate the null distributions. The permutation test for interaction is complicated, because there is no exact permutation test for such a purpose [43]. We therefore must consider an approximate permutation method for testing an interaction term in a crossed fixed/mixed model [44,45].

Permutation approaches developed previously focused on a single ANOVA model. In the typical gene expression study, thousands of ANOVA models are considered simultaneously. The additional complexity of the shrinkage *F*-like statistics indicates that Monte Carlo studies are needed to investigate the performance of residual permutation and raw data permutation, with restrictions or not, in a gene-expression analysis. The choice of permutation procedures is critical for assessing the performance of a test statistic.

For all the modified *F*-like statistics presented in the previous section, the null distributions can only be approximated empirically, but permutation procedures can be used to find the approximate null distribution of all the *F *and *F*-like statistics. The important issues in performing a permutation analysis include the choice of the exchangeable units under the null hypothesis, the choice of using restricted permutation or not, and the choice of residual permutation or raw data permutation. These choices influence the power of a test statistic.

Residual permutation using residuals from a reduced model and unrestricted raw data permutation can be used to approximate the null distribution of a statistic for testing an interaction term [44]. When using *F*_1 _to test an interaction term in a single ANOVA model, the residual permutation leads to a more powerful test than unrestricted raw data permutation [44]. However, in gene expression analysis, thousands of gene-specific ANOVA models are simultaneously considered, and for a particular gene-specific ANOVA model, information from other gene-specific ANOVA models is used to construct the shrinkage error estimate. Hence, both residual permutation and raw data permutation were investigated. Furthermore, both restricted and unrestricted permutations were studied, because the permutation units are exchangeable only within each particular group when within-gene heteroscedasticity is present across those subgroups.

## Results

The properties of this shrinkage estimator are compared with those of other existing *F *and *F*-like statistics that have been proposed and described in the "Shrinkage *F*-like statistics" section.

### Simulation studies

The purpose of these simulation studies was to compare the performances of *F*_1_, *F*_2_, *F*_3_, *F*_*Cui*_, *F*_*Gen*_, *F*_*Gen-gene*_, and *F*_*Gen-grp *_in terms of type I error and power and to compare the results of a particular *F*-like statistic using four different permutation strategies: restricted/unrestricted residual permutation and restricted/unrestricted raw data permutation.

In these simulation studies, 100 genes with two probes for each gene and three replicates from each of two lines were simulated to mimic a split-plot design in a general oligonu-cleotide microarray experiment. The gene-specific ANOVA model in which data were generated from the model, *y*_*plr *_= *P*_*p *_+ *L*_*l *_+ *RL*_*rl *_+ *PL*_*pl *_+ ϵ_*plr*_, w*p *= 1, 2, *l *= 1,2, *r *= 1,2,3, where *P, L, RL*, and *PL *represent probe, line, replicates from a particular line, and the interaction between probe and line, respectively.

Replicates were nested within each line, and *RL *is usually treated as a random effect during the model-fitting procedure, which results in a correlation between probes from the same biological sample. In the simulated data sets, the correlation between genes was 0. As many as 900 simulation runs were carried out to compare the performances of *F*_1_, *F*_2_, *F*_3_, *F*_*Cui*_, *F*_*Gen*_, *F*_*Gen-gene*_, and *F*_*Gen-grp *_based on different permutation procedures. The four permutations tested were unrestricted residual permutation, restricted residual permutation with respect to each line, unrestricted raw data permutation, and restricted raw data permutation with respect to each line. The residuals permuted were from a reduced fixed model with fixed effects for only line and probe.

**Figure 1:**
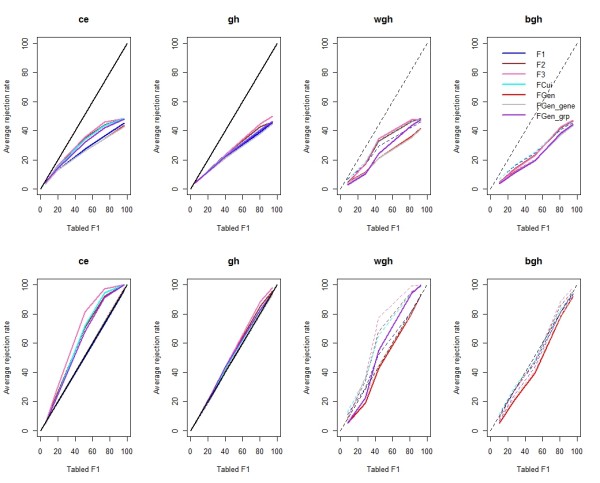
**The comparison of power curves of all *F*-like test statistics**. The x-axis is the average power from analyzing 900 simulated data sets using *F*_1 _with tabled *p*-values. The y-axis is the estimated powers using empirical gene-specific null distributions from 1,000 residual permutations. The upper four plots show the results with restricted residual permutation, while the lower four plots show the results from unrestricted residual permutation. The solid line indicates the empirical average CWER of a statistic is at the prespecified level, and the dashed line shows an inflated empirical average CWER."ce," all genes have common error; "gh," only between-gene heteroscedasticity exists; "wgh," only within-gene heteroscedasticity exists; "bgh," both between-gene and within-gene heteroscedasticity exist.

**Figure 2:**
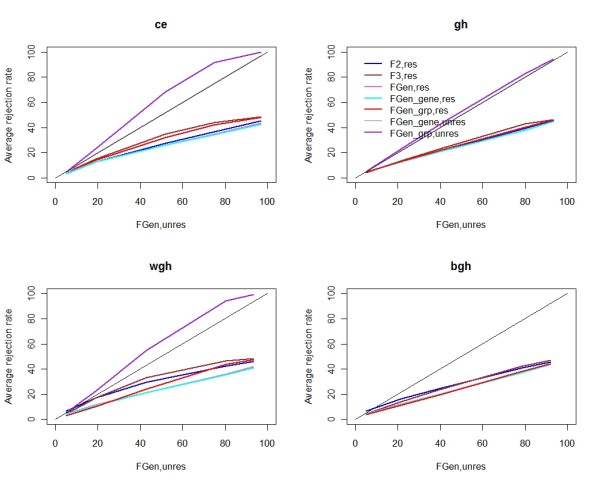
**The comparison of power curves of *F***_***Gen ***_**from unrestricted residual permutation versus other *F*-like test statistics**. Only results from permutation combinations that can control prespecified CWER are used in this figure. The x-axis is the average power after analyzing 900 simulated data sets using *F*_*Gen *_and 1,000 unrestricted residual permutations. The y-axis is the estimated power from other *F*-like test statistics and empirical gene-specific null distributions based on the appropriate permutation. The solid black line corresponds to *F*_*Gen *_with unrestricted permutation, and this test always controls prespecified CWER."ce," all genes have common error; "gh," only between-gene heteroscedasticity exists; "wgh," only within-gene heteroscedasticity exists; "bgh," both between-gene and within gene heteroscedasticity exist; "res," restricted permutation; "unres," unrestricted permuation.

Two types of data were simulated: null cases and cases with a probe by line interaction at a range of degrees. Null cases included: *null-ce*, all probe-level expression values were simulated from the standard normal distribution; *null-gh*, the gene-specific error variances were simulated from the log-normal distribution with mean log at 0 and standard deviation at 2, mimicking the general heteroscedastic error distribution in typical datasets; *null-wgh*, all genes had the same error structures and the residual error variance of line 1 was 100 times that of line 2; *null-bgh*, simulated data were modified from *null-gh*, with the variance of line 1 multiplied by 100. Correspondingly, *ce, gh, wgh*, and *bgh *in Figures 1 and 2 were simulated by adding interaction terms to *null-ce, null-gh, null-wgh*, and *null-bgh*. Quantitative interaction was assumed and the differences in the opposite direction were set to make the detection powers for an interaction term based on traditional F-statistics and tabled p-values range from 0.05 to 0.95.

Tables 1 and 2 show the results from 900 simulation runs using raw data permutation and residual permutation, respectively. Data in Table 1 suggest that when both types of gene heteroscedasticity exist, the unrestricted raw data permutation had a greater average comparison-wise error rate (CWER) than residual permutation. Raw data permutation with restriction can control prespecified CWER im all cases. In Table 2, for the common error cases, all test statistics had the prespecified CWER from both restricted and unrestricted residual permutation. When within-gene heteroscedasticity existed, *F*_1 _and *F*_*Cui *_had inflated CWER from both two residual permutation tests. Restricted residual permutation reduces, but does not solve, this problem. For *F*_2 _and *F*_3_, only the restricted residual permutation could control the prespecified CWER. For *F*_*Gen*_, *F*_*Gen-gene*_, and *F*_*Gen-grp*_, restricted residual permutation gave conservative results in terms of having CWER smaller than the prespecified level. When the shrinkage target is correctly set, unrestricted residual permutation controls the nominal CWER. As expected, only *F*_*Gen *_coupled with unrestricted residual permutation could be used for all cases, because the CWER was always less than the nominal level.

**Table 2:** Results from residual permutation

Restricted?	Data set	*F*_1_	*F*_2_	*F*_3_	*F*_*Cui*_	*F*_*Gen*_	*F*_*Gen-gene*_	*F*_*Gen-grp*_
YES	null-ce	4.59(0.07)	4.15(0.08)	3.63(0.14)	4.09(0.09)	4.55(0.07)	3.23(0.06)	4.44(0.08)
	null-gh	4.57(0.07)	4.1(0.13)	3.95(0.16)	4.49(0.07)	4.6(0.07)	4.61(0.07)	4.38(0.07)
	null-wgh	6.74(0.08)	4.33(0.08)	3.51(0.14)	6.49(0.09)	4.38(0.07)	4.2(0.07)	2.78(0.09)
	null-bgh	6.74(0.08)	4.35(0.16)	4.07(0.19)	6.58(0.08)	4.36(0.07)	4.16(0.07)	3.64(0.07)

NO	null-ce	5.1(0.07)	4.99(0.08)	4.5(0.08)	4.59(0.08)	4.99(0.07)	4.1(0.07)	4.68(0.07)
	null-gh	5.1(0.07)	4.83(0.1)	4.59(0.11)	5.08(0.07)	4.99(0.07)	5.01(0.07)	4.95(0.07)
	null-wgh	10.75(0.09)	8.46(0.09)	7.6(0.09)	12.37(0.11)	5.03(0.08)	6.43(0.08)	4.93(0.08)
	null-bgh	10.75(0.1)	8.38(0.17)	8.07(0.19)	10.79(0.1)	5.02(0.08)	6.38(0.08)	6.73(0.08)

CWER obtained from 1,000 permutations with the nominal significance level setting at 0.05, with standard errors in parentheses. Nine hundred simulation runs were performed to get empirical average CWER of all types of *F*-like test statistics.

Further simulations to compare the rejection rates were conducted. Only results from residual permutation are shown because it was found that raw data permutation was less powerful than residual permutation. This is consistent with the findings of Anderson and Ter Braak [44]. Figure 1 shows the estimated average null hypothesis rejection rate curves from all *F*-like statistics and both restricted and unrestricted residual permutation procedures. The x-axis represents the average null hypothesis rejection rate using *F*_1 _and the tabulated *p*-values. The solid line shows that the corresponding statistic controls the prespecified CWER, and the dashed line shows that the corresponding CWER was inflated. In general, restricted residual permutation is less powerful than unrestricted residual permutation. For example, the power of all statistics from unrestricted residual permutation almost doubled in some cases where heteroscedasticity existed.

When the common error assumption is valid, *F*_3 _is obviously the most powerful test and the prespecified CWER is controlled. All other *F*-like statistics performed very similarly in this case. When the shrinkage target was correctly set, the resultant test statistic was the most powerful one. For example, when there was only within-gene heteroscedasticity, *F*_*Gen-grp *_was more powerful than *F*_*Gen *_and *F*_*Gen-gene *_based on either restricted or unrestricted residual permutation. The rejection rate comparison of statistically valid test statistics is further illustrated in Figure 2, where the x-axis is the average rejection rate from using *F*_*Gen *_and unrestricted residual permutation. Figure 2 clearly shows that unrestricted residual permutation is more favorable in terms of power. *F*_*Gen-grp *_appears to be more powerful than *F*_*Gen*_, but when both types of gene heteroscedasticities occur, *F*_*Gen grp *_has inflated CWER.

### Drosophila data

The data used in this study are from a gene expression comparison study between *D. melanogaster *and *D. simulans *[46]. Expression of 10 genotypes of each species was measured in male flies. In *D. simulans*, each genotype was measured separately, and in *D. melanogaster*, a pool of 10 genotypes was measured. All genotypes (individual or pooled) were independently isolated and hybridized three times. The goal of the original study was to provide a genome-wide approach to identifying candidate genes potentially responsible for adaptation and speciation in *D. simulans *and *D. melanogaster*. In this study, we focus on identifying sequence differences between genotypes in *D. simulans *based on hybridization profiles. Within-gene heteroscedasticity is expected because the genotypes come from different lines. The proposed generalized shrinkage *F*-like test statistics *F*_*Gen*_, *F*_*Gen-gene*_, and *F*_*Gen-grp *_were compared with *F*_2_, *F*_3 _with restricted residual permutation, which could control prespecified CWER for any variance structure in simulation studies. Furthermore, Smyth's moderated F-test statistic [25] without multiple testing adjustment and controlling the false discovery rate (FDR) at 5% were used for comparison. As the main interest is in sequence difference, the focus is on the test of interaction between line and probe. The split plot model described above is used. SAS program codes are included in the additional files (additional file 1 and additional file 2).

The Drosophila genome has been fully sequenced and both SNPs and indels can cause a significant interaction term. Thus, the false positive rate and detection power based on SNP/indel sequence information can be calculated for a subset of the data. In the data set, there were 10 lines from *D. simulans *and three replicates from each line. Each probe set had 14 probes. The 1,285 probesets containing all "good" probes were selected. A "bad" probe's sequence satisfies one or more of the following criteria: it matches the *D. simulans *genome multiple times; it cannot be mapped to the flybase 4.2.1 genome; or, it has no information, such as hitting outside an exon, hitting a poorly aligned region, or hitting a region lacking a sequence. SNP or indel information could be determined in 777 probesets. For this data set, there was a high degree of within-gene heteroscedasticity: about 22.3% of the probe sets had a difference in line-specific residual variance estimates as large as or more than a 10-fold change. Therefore, as suggested by the conclusions from simulation studies, unrestricted residual permutation and restricted residual permutation were used for generalized shrinkage *F*-like test statistics (*F*_*Gen*_, *F*_*Gen-gene*_, *F*_*Gen-grp*_) and restricted residual permutation was used for statistics (*F*_2_, *F*_3_). The results are shown in Table 3. Consistent with the findings from the simulation studies, *F*_*Gen *_had about 30% more detecting power by valuing the within-gene heteroscedasticity than the other *F*-like test statistics (*F*_2_, *F*_3_). The false discovery rate of *F*_*Gen *_was slightly higher than that of *F*_2_, *F*_3_. *F*_*Gen-gene *_and *F*_*Gen-grp *_performed similarly to *F*_*Gen*_. Both of Smyth's moderated F-test statistic without multiple testing adjustment and with FDR set at 5% for multiple testing adjustment detected more SNPs and indels but at the expense of a greater FDR than *F*_*Gen*_.

**Table 3:** Probe sets with significant line*probe terms found by F-like test statistics and appropriate residual permutation procedures and Smyth's moderated F-test statistic

Test statistic	Restricted permutation?	Number of probe sets found	True false discovery rate	Power
*F*_2_	Yes	124	22.6%	12.4%
*F*_3_	Yes	187	29.4%	17.1%
*F*_*Gen*_	No	453	29.5%	41.1%
*F*_*Gen-gene*_	No	455	28.8%	41.7%
*F*_*Gen-grp*_	No	474	28.9%	43.4%
*F*_*Gen*_	Yes	136	24.3%	13.3%
*F*_*Gen-gene*_	Yes	122	22.1%	12.2%
*F*_*Gen grp*_	Yes	116	21.5%	11.7%
*moderatedF *- 1	N/A	535	34.1%	75.5%
*moderatedF *- 2	N/A	813	34.4%	68.8%

The CWER was set to 0.05. Gene-specific cutoff values were obtained from 1,000 permutations. "moderated F-1" and "moderated F-2" represent results from using moderated F statistic without any multiple testing adjustment and setting FDR to 5%.

## Discussion

For gene expression analysis, ANOVA models have been a popular modeling technique. Based on ANOVA models, flexible shrinkage *F*-like test statistics were developed to account for both the within-gene and between-gene heteroscedasticities. The emphasis here is on testing an interaction term, as this case is of increasing interest to biologists, and there is no clear existing theory on the most powerful, valid approach for such statistics. For all *F*-like statistics studied here, their null distributions were approximated empirically through permutations. Four different permutation procedures were investigated for eight different *F*-like statistical tests of the interaction term.

As expected, we found that when an error estimator overshrinks, the resulting *F*-like statistic cannot control the prespecified CWER. For example, *F*_*Gen-gene *_is an over-shrinkage error estimator when there is within-gene heteroscedasticity. As a result, compared with generalized shrinkage *F*-like statistics, it is not valid when within-gene heteroscedasticity exists. Undershrinkage is also important, as it will lead to a conservative test and lower power. This is clearly demonstrated when the common error can be assumed and the most powerful valid test is *F*_*Gen-grp*_.

The most striking result was the impact of the permutation procedures. Although this was not completely unexpected [43-45], the effect of the permutation procedures is dramatic and worthy of special attention. Unrestricted raw data permutation could not control prespeci-fied CWER when there was within-gene heteroscedasticity. Restricted raw data permutation could be used, but it was less powerful than residual permutation. Also consistent with findings from Anderson and Ter Braak [44], restricted permutations are less powerful than unrestricted permutations. However, unrestricted permutations are valid only for a common error and when between-gene heteroscedasticity exists for our proposed shrinkage statistics; they are not valid in combination with *F*_2_, *F*_3_, or *F*_*Cui*_. For *F*_*Gen-grp*_, the unrestricted permutation can also be used in cases having within-gene heteroscedasticity, while only *F*_*Gen *_is valid with unrestricted permutation in all cases in terms of controlling prespecified CWER. Interestingly, the power gain from using the correct shrinkage target *F*_*Gen-grp *_rather than *F*_*Gen *_is far less than that of using unrestricted permutation. The result is that *F*_3 _is never the most powerful choice when testing an interaction term.

The correct shrinkage target can lead to the most powerful test statistic. As one of the reviewers suggested, a statistical test may be applied to help pick the best shrinkage target before obtaining shrinkage error estimates. However, this extra testing step may inflate the CWER of the test statistic when there is gene heteroscedasticity. For example, when there are both types of gene heteroscedasticities, it is possible that the above test suggests only within-gene heteroscedasticities exist, and *F*_*Gen-grp *_is shown to inflate the CWER. There is minimal penalty to using the shrinkage estimator we propose, so we recommend setting the shrinkage target in the full space spanned by group and gene and using unrestricted permutation to compensate for the possible power loss in fewer degrees of freedom left for estimating the errors.

## Conclusions

The proposed generalized shrinkage *F*-like statistic with shrinkage targets located in a space spanned by gene and another group, *F*_*Gen*_, with unrestricted residual permutation is always valid in terms of having a prespecified CWER. This statistic has reasonable power in most cases; thus, it is generally recommended to be applied to test an interaction term in the analysis of real gene expression data.

## List of abbreviations

CWER: comparison-wise error rate; FDR: false discovery rate; indel: insertion and deletion; SNP: single nucleotide polymorphism;

## Authors' contributions

All authors contributed to the design of the overall strategy. JY carried out all the analysis and drafted the manuscript. LMM and GC helped to draft and finalize the manuscript. All authors read and approved the final manuscript.

## Supplementary Material

Additional file 1 **SAS program code 1**. SAS program code for analyzing the real data set using residual permutation without restriction. Click here for file

Additional file 2 **SAS program code 2**. SAS program code for analyzing the real data set using residual permutation with restriction. Click here for file
